# Synthetic Biology Approaches to Engineer *Saccharomyces cerevisiae* towards the Industrial Production of Valuable Polyphenolic Compounds

**DOI:** 10.3390/life10050056

**Published:** 2020-05-02

**Authors:** João Rainha, Daniela Gomes, Lígia R. Rodrigues, Joana L. Rodrigues

**Affiliations:** Centre of Biological Engineering, University of Minho, 4710-057 Braga, Portugal; joao.rainha@ceb.uminho.pt (J.R.); danielafcr.gomes@gmail.com (D.G.); lrmr@deb.uminho.pt (L.R.R.)

**Keywords:** polyphenols biosynthesis, synthetic biology, *Saccharomyces cerevisiae*, metabolic engineering, heterologous production

## Abstract

Polyphenols are plant secondary metabolites with diverse biological and potential therapeutic activities such as antioxidant, anti-inflammatory and anticancer, among others. However, their extraction from the native plants is not enough to satisfy the increasing demand for this type of compounds. The development of microbial cell factories to effectively produce polyphenols may represent the most attractive solution to overcome this limitation and produce high amounts of these bioactive molecules. With the advances in the synthetic biology field, the development of efficient microbial cell factories has become easier, largely due to the development of the molecular biology techniques and by the identification of novel isoenzymes in plants or simpler organisms to construct the heterologous pathways. Furthermore, efforts have been made to make the process more profitable through improvements in the host chassis. In this review, advances in the production of polyphenols by genetically engineered *Saccharomyces cerevisiae* as well as by synthetic biology and metabolic engineering approaches to improve the production of these compounds at industrial settings are discussed.

## 1. Plant Polyphenols

The secondary metabolism refers to the metabolic pathways that lead to the compounds that are not involved in the processes of growth, development and reproduction [1]. Plants produce diverse secondary metabolites including phenolics, terpenes and steroids, and alkaloids that are stored in specific organelles at low concentrations [2]. These compounds are responsible for the plant adaptation against biotic and abiotic stresses and have a wide range of applications as pharmaceuticals, cosmetics and fine chemistry, among others [2,3].

**Figure 1:**
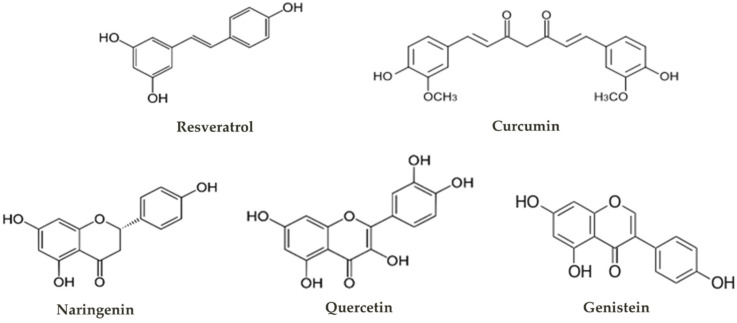
Chemical structure of some polyphenols.

Phenolics are a large group of plant secondary metabolites characterized by containing at least one hydroxylated aromatic ring. This group includes the hydroxycinnamic acids, such as *p*-coumaric acid, caffeic acid and ferulic acid. These compounds are the precursors of complex polyphenolic molecules whose expected market value is USD 2.26 billion by 2027 [4]. Polyphenols can be divided in flavonoids, stilbenoids, curcuminoids, coumarins, polyphenolic amides and lignans. Some of these compounds are more documented and studied, namely flavonoids, stilbenoids and curcuminoids (Figure 1). For example, flavonoids (expected market value: 1.26 billion USD by 2026 [5]) are the most studied subgroup with more than 4000 varieties identified. Regarding stilbenoids, the resveratrol (expected market value: 149.43 million USD by 2027 [6]) found in grapes is the most well documented. Curcuminoids are plant-specific polyphenols produced by Zingiberates order species such as the *Curcuma longa* and are widely used as dietary spice, and curcumin is the one most explored due to its suggested superior biological activities (expected market value: 151.9 million USD by 2027 [7]).

### 1.1. Biological Activities

Plant polyphenols have shown numerous biological activities [8,9,10,11,12,13] and, in recent decades, several epidemiological studies were carried to elucidate their therapeutic effects against several human diseases or conditions. The most reported characteristic for polyphenols is their capacity to scavenge reactive oxygen species (ROS) and to inhibit other oxidative damages such as lipid oxidation, hydroperoxide formation and DNA damage in the human body. At the cellular level, polyphenols may act as antioxidants through their effect on plasma membranes, transcription factors and in the enzyme activity. This chemical reactivity is conferred by the hydroxyl groups attached to the benzene group which confers a stronger acidic character to phenol [14]. For example, the antioxidant activity of rosmarinic acid and salvionic acid were determined using 2,2-diphenyl-1-picrylhydrazyl radical scavenging by Zhang et al. [15] and these two polyphenols were classified as strong antioxidant molecules. More specific tests have already been used to prove the antioxidant effect of polyphenols in the human body. For instance, Gallardo et al. [16], tested the in vitro protective effect of resveratrol against the oxidation caused by hypochlorous acid (HClO) in red blood cells, using thermal fluctuation spectroscopy to measure the fluctuations in the cell’s membrane. The researchers suggested that the treatment with resveratrol resulted in a protective effect against oxidation since the deformation of the red blood cells decreased when the resveratrol treatment was applied. Moreover, in vivo tests have also been performed. García-Alonso et al. [17] evaluated the short-term effect of phenolic rich juice on the antioxidant status of healthy human subjects. The results indicated that polyphenols were able to bind the lipid content of serum and consequently reduce lipid peroxidation.

In addition, polyphenols have shown anticancer effects against various carcinomas. These compounds have several mechanisms of action targeting from DNA mutations to metastasis and apoptosis processes [13]. Moreover, other therapeutic effects have been reported such as anti-inflammatory, anti-Alzheimer, anti-Parkinson, anti-diabetic or anti-HIV effects. Table 1 summarizes examples of biological activities and the respective mechanisms of action of some polyphenols.

### 1.2. Polyphenol Biosynthesis

**Figure 2:**
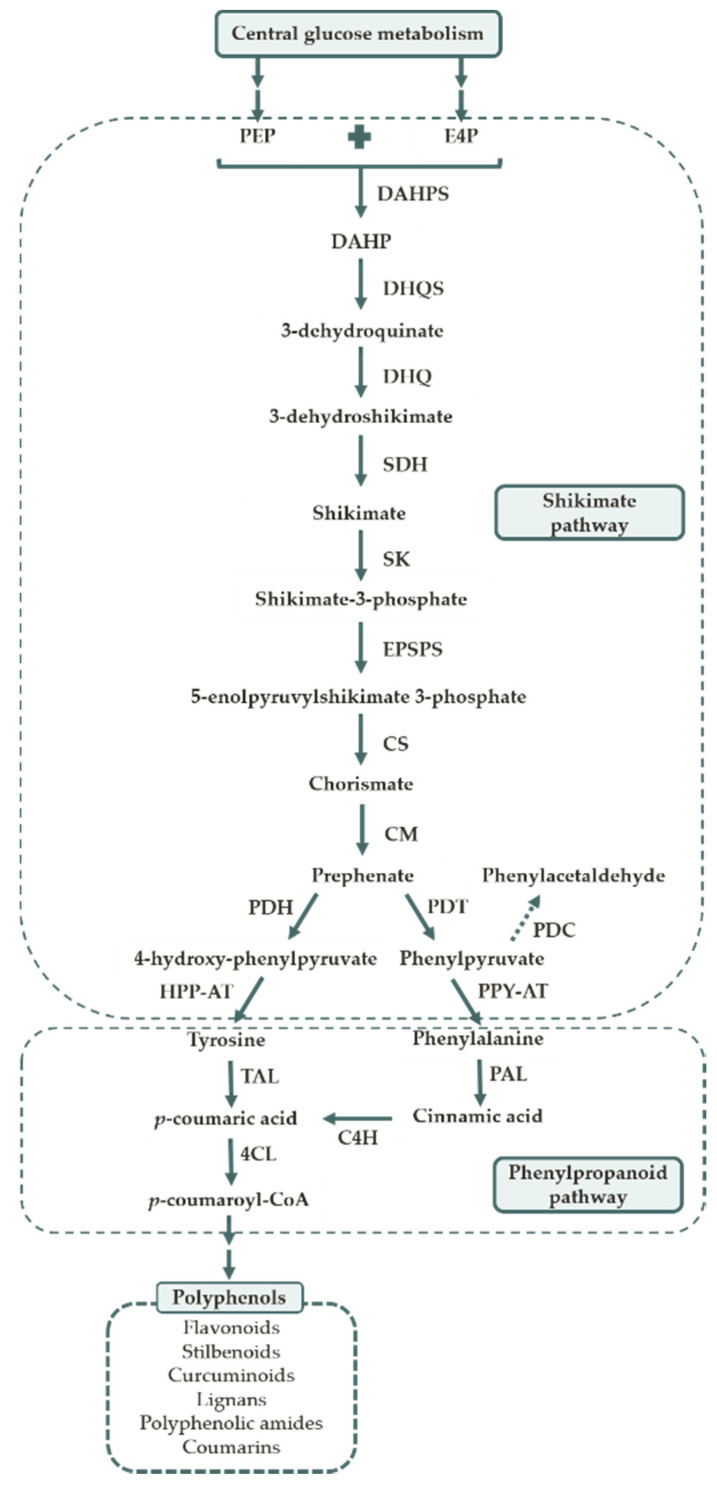
Pathways involved in the polyphenol biosynthesis. 4CL—4-coumarate-CoA ligase; C4H—cinnamate-4-hydroxylase; CM—chorismate mutase; CS—chorismate synthase; DAHP—3-deoxy-D-arabino-heptulosonate-7-phosphate; DAHPS—3-deoxy-D-arabino-heptulosonate-7-phosphate synthase; DHQ—3-dehydroquinate dehydratase; DHQS—3-dehydroquinate synthase; E4P—D-erythrose-4-phosphate; EPSPS—5-enolpyruvylshikimate 3-phosphate synthase; HPP-AT—4-hydroxyphenylpyruvate aminotransferase; PAL—phenylalanine ammonia lyase; PDC—phenylpyruvate decarboxylase; PDH—prephenate dehydrogenase; PDT—prephenate dehydratase; PEP—Phosphoenolpyruvic acid; PPY-AT—phenylpyruvate aminotransferase; SDH—shikimate dehydrogenase; SK—shikimate kinase; TAL—tyrosine ammonia lyase.

There are two biosynthetic pathways involved in the polyphenol biosynthesis in plants, namely the shikimate pathway and the phenylpropanoid pathway. A schematic representation of the phenylpropanoid biosynthesis is illustrated in Figure 2. 

The shikimate pathway consists of seven sequential enzymatic steps. The first one is the condensation of phosphoenolpyruvic acid (PEP) and D-erythrose-4-phosphate (E4P). These two phosphorylated active compounds are derived from the glycolytic pathway and the pentose phosphate cycle, respectively. The last reaction of this pathway is catalyzed by chorismate mutase (CM) to form chorismate. Then, chorismate is converted into the aromatic amino acids, phenylalanine and tyrosine, by successive reactions [35].

**Figure 3:**
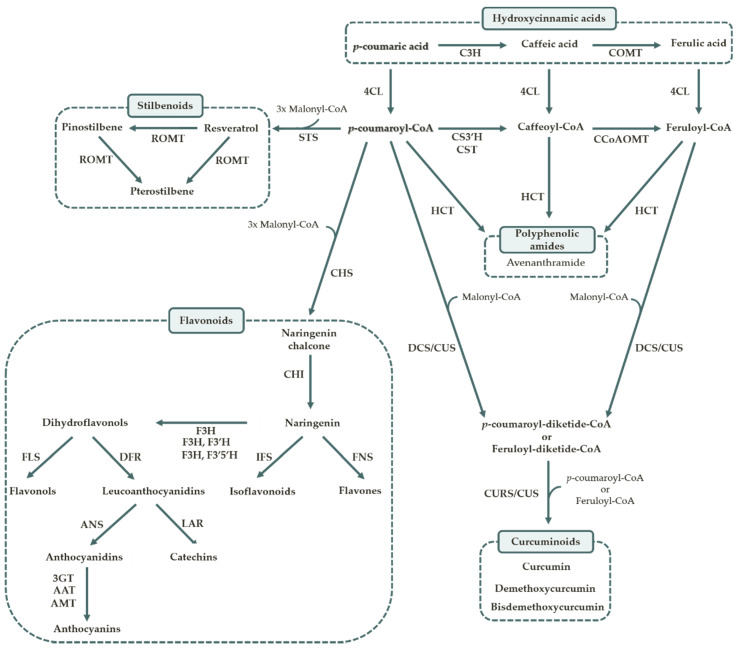
Steps involved in the hydroxycinnamic acids, flavonoids, stilbenoids, polyphenolic amides and curcuminoids biosynthesis from *p*-coumaric acid. 3GT—anthocyanidin 3-O-glycosyltransferase; 4CL—4-coumarate-CoA ligase; AAT—anthocyanin acyltransferase; AMT—anthocyanin methyltransferase; ANS—anthocyanidin synthase; C3H—4-coumarate 3-hydroxylase; COMT—caffeic acid 3-O-methyltransferase; CCoAOMT—caffeoyl-CoA 3-O methyltransferase; CHI—chalcone isomerase; CHS—chalcone synthase; CS3′H—*p*-coumaroyl 5-O-shikimate 3′-hydroxylase; CST—*p*-coumaroyl shikimate transferase, CURS—curcumin synthase; CUS—curcuminoid synthase; DCS—diketide-CoA synthase; DFR—dihydroflavonol 4-reductase; F3H—flavanone 3-hydroxylase; F3′H—flavonoid 3′-hydroxylase; F3′5′H—flavonoid 3′5′-hydroxylase; FLS—flavonol synthase; FNS—flavone synthase; HCT—hydroxycinnamoyl-CoA: Shikimate/quinate hydroxycinnamoyl transferase; IFS—isoflavone synthase; LAR—leucoanthocyanidin 4-reductase; ROMT—resveratrol O-methyltransferase; STS—stilbene synthase.

Subsequently, the phenylpropanoid pathway channels the carbon flow from amino acid metabolism to different branch pathways of secondary metabolism which include the synthesis of the diverse polyphenols. Phenylalanine ammonia lyase (PAL) is the first enzyme of this pathway and it represents a branch point between primary and secondary metabolism. It catalyzes the formation of cinnamic acid. Then, cinnamic acid is converted to *p*-coumaric acid by cinnamate-4-hydroxylase (C4H). Alternatively, tyrosine can also be used as an initial substrate of the pathway. In this reaction, tyrosine is directly converted into *p*-coumaric acid by tyrosine ammonia lyase (TAL). Afterwards, *p*-coumaric acid can be metabolized in two different ways. It may be converted to other hydroxycinnamic analogues or it can be activated to form the coenzyme A (CoA) ester (Figure 3). The production of *p*-coumaroyl-CoA is catalyzed by 4-coumarate-CoA ligase (4CL). This enzyme acts in multiple substrates, activating also the other analogues. After activation, *p*-coumaroyl-CoA can also be converted into the other activated analogues, caffeoyl-CoA and feruloyl-CoA. At this point the polyphenol biosynthesis diverges and the enzymes involved depend on the type of polyphenol produced.

Afterwards, some of the reactions are catalyzed by a group of enzymes called type III polyketide synthase (PKS) and despite their structural simplicity, type III PKSs produce a wide array of compounds [36]. There are several varieties and each one catalyzes the formation of a different polyphenol or a polyphenol group. Type III PKSs use the activated CoA esters (such as *p*-coumaroyl-CoA) as starter units and malonyl-CoA molecules as extender units [36]. For instance, the first step in flavonoid biosynthesis is performed by chalcone synthase (CHS). This enzyme catalyzes the formation of naringenin chalcone from *p*-coumaroyl-CoA and three molecules of malonyl-CoA. Afterwards, naringenin chalcone is converted into naringenin by chalcone isomerase (CHI). Subsequent reactions are responsible for the synthesis of other specific flavonoids resulting in the chemodiversity of these compounds [37]. Other type III PKSs include stilbene synthase (STS), diketide-CoA synthase (DCS), curcuminoid synthase (CUS) and curcumin synthase (CURS). In the polyphenolic amides pathway, the CoA esters are converted into amides such as avenanthramide by hydroxycinnamoyl-CoA:shikimate/quinate hydroxycinnamoyl transferase (HCT), and there is no proof so far that malonyl-CoA acts as an extender substrate [38]. Regarding coumarins, they can also originate from the three CoA esters by the action of *p*-coumaroyl-CoA 2′-hydroxylase (C2′H) [39]. The lignans pathway starts through the dimerization of two molecules of coniferyl alcohol that are produced after two enzymatic steps from feruloyl-CoA [40]. As far as we know, there have been no studies reporting the heterologous production of coumarins, curcuminoids and lignans in *S. cerevisiae*.

### 1.3. Polyphenol Heterologous Production 

Given all the benefits of plant polyphenols, the production of larger quantities of these compounds is important to satisfy the demand and to enable a deeper study of their health-promoting effects. However, plant secondary metabolites are accumulated in low quantities and vary widely among different plant species and tissues. Moreover, the availability of these compounds is affected by seasonal and geographical variations. Their extraction from the native plants is characterized by low yields and complex downstream processes [41]. Besides, many plants containing high-value compounds are difficult to cultivate, and over harvesting may result in their depletion. The chemical synthesis could represent a fast way to synthesize them. However, it requires the use of expensive precursors, toxic catalysts and extreme reaction conditions. Therefore, the process is neither amenable for a large-scale production nor eco-friendly [42].

The heterologous production in plants is an alternative approach to obtain these valuable compounds. This approach has the advantage of requiring the introduction of less genes since the phenylpropanoid pathway is already present. However, plants possess competitive pathways that could greatly interfere with the polyphenol biosynthesis. Moreover, these heterologous plants also take a long time to grow and the growth is dependent on weather conditions and on fertile land availability. In addition, they are difficult to genetically manipulate. The use of plant cell cultures, although being a way to overcome those limitations, has also some disadvantages such as heterogeneity, variable yields, low growth rates, unstable cultures, susceptibility to stresses and aggregation [43]. The reconstruction of the biosynthetic pathways in microorganisms could be an alternative method to meet the industrial requirements for polyphenol production. The use of engineered microorganisms represents a promising strategy towards a more rapid, inexpensive and green process. Genetically modified microorganisms have several advantages since they can grow in inexpensive substrates, are easier to manipulate and have fast production cycles allowing a faster and larger production. The use of microorganisms allows large-scale fermentations and the downstream purification processes become easier due to the absence of competing pathways [44]. Moreover, microorganisms have a primary metabolism similar to plants being able to provide the aromatic amino acids and malonyl-CoA as precursor molecules. Therefore, the use of microbial cell factories appears to be an ideal solution to industrially produce plant therapeutic polyphenols.

The most commonly used microorganisms for metabolic engineering purposes are the bacteria *Escherichia coli* and the yeast *Saccharomyces cerevisiae*. These organisms are very well characterized, easy to grow, manipulate and scale-up. *S. cerevisiae* is a key laboratorial and industrial microorganism and represents an excellent chassis for metabolic engineering and synthetic biology approaches. *S. cerevisiae* was the first eukaryotic organism whose genome was fully sequenced [45]. This microorganism has been extensively engineered to produce a wide variety of compounds and proteins. In addition, the knowledge on its physiology, genetics and fermentation patterns is vast, similarly to *E. coli*. However, it presents some unique characteristics over the bacteria to produce plant compounds. Being a eukaryotic organism, it is capable of post-translational modifications such as glycosylation and it possesses intracellular compartments similarly to plants. Moreover, *S. cerevisiae*, unlike *E. coli*, has a food-grade status allowing its use in human nutrition and pharmaceuticals [45]. In recent years, many efforts have been developed to heterologously produce hydroxycinnamic acids and polyphenols using *S. cerevisiae* (Table 2).

## 2. Genetic Engineering of *Saccharomyces Cerevisiae*

The design, construction and optimization of biochemical pathways in *S. cerevisiae* is mandatory to achieve high amounts of heterologous compounds. In the next sections, examples of heterologous production of polyphenolic compounds and respective optimizations in *S. cerevisiae* using synthetic biology approaches will be described. 

### 2.1. Synthetic Biology Tools for Genome Engineering

*S. cerevisiae* is easily manipulated through molecular and synthetic biology techniques. The classical restriction–ligation-based cloning is being gradually replaced by new methods for the rapid design and construction of large biochemical pathways like the “DNA assembler” [46]. The excellent homologous recombination (HR) capability of this microorganism facilitates the use of these techniques. Two synthetic biology tools that are used to genetically engineer heterologous biosynthetic pathways in *S. cerevisiae* genome are discussed below.

**Table 2:** Production of hydroxycinnamic acids and polyphenols in *Saccharomyces cerevisiae*. The genes, genetic systems, chassis, chassis modifications, substrates, conditions and titers obtained.

Phenolic Compound	Genes ^1^	Genetic System	Chassis/Chassis Modification ^2^	Substrate ^3^	Conditions	Titer (mg/L)	Reference
**Coumaric acid**	*Fj*TAL	One copy genome integration	*S. cerevisiae* CEN.PK102-5B/Aromatic amino acids overproducing strain	Synthetic fed-batch medium	Microtiter plates	1900	[47]
**Coumaric acid**	*Fj*TAL	One copy genome integration	*S. cerevisiae* CEN.PK102-5B/Knockout of the downregulated transporter TAT1	Glucose (10 g/L)	Fed-batch	2400	[48]
**Coumaric acid**	*Fj*TAL, xylose utilization genes	One copy genome integration	*S. cerevisiae* CMB.GS010/Aromatic amino acids overproducing strain	Xylose (15 g/L)	Batch bioreactor	242	[49]
**Coumaric acid**	*AtPAL, AtC4H*	One copy genome integration	*S. cerevisiae* IMX/Aromatic amino acids overproducing strain; Overexpression of CYB5 and expression of *At*CPR to enhance cytochrome P450 activity	Glucose (20 g/L)	Fed-batch	12500	[50]
**Caffeic acid**	*Rt*TAL, *Pa*HpaB, *Se*HpaC	Episomal plasmid	*S. cerevisiae* BY4741/-	Tyrosine (0.5 g/L)	Shake flask	289.4	[51]
**Caffeic acid**	*Rc*TAL, *At*C3H, *At*CPR	One copy genome integration	*S. cerevisiae* BY4742/Aromatic amino acids overproducing strain; Expression of *At*CPR to enhance cytochrome P450 activity	Glucose (40 g/L)	Shake flask	11.4	[52]
**Resveratrol**	*Ph*4CL216, *Vv*VST1	Episomal plasmid	*S. cerevisiae* FY23/-	Coumaric acid (10 mg/L)	Shake flask	0.0014	[53]
**Resveratrol**	*Nt*4CL2, *Vv*STS	One copy genome integration	*S. cerevisiae* CEN-PK113-3B/-	Coumaric acid (820 mg/L)	Shake flask	5.8	[54]
**Resveratrol**	*At*4CL1, *Vv*STS	Episomal plasmid	*S. cerevisiae* strain isolated from a Brazilian sugar cane plantation.	Coumaric acid (2.46 g/L)	Shake flask	391	[55]
**Resveratrol**	*At*4CL1, *Ah*STS	Episomal plasmid	*S. cerevisiae* W303-1A/PAD knock-out	Coumaric acid (16 mg/L)	Shake flask	3.1	[56]
**Resveratrol**	*At*4CL1 and *Vv*STS fusion protein	One copy genome integration	*S. cerevisiae* WAT11/Expression of *araE* transporter	Coumaric acid (10 mg/L) every 24 h	Shake flask	2.3	[57]
**Resveratrol**	*Rt*PAL, *At*C4H, *At*4CL1, *Ah*STS	Episomal plasmid	*S. cerevisiae* W303-1A/Overexpression of ACC1	Tyrosine (2.17 g/L)	Batch bioreactor	5.8	[58]
**Resveratrol**	*At*4CL1 and *Vv*STS synthetic scaffold	Episomal plasmid	*S. cerevisiae* WAT11/-	Coumaric acid (20 mg/L)	Shake flask	14.4	[59]
**Resveratrol**	*Ha*TAL, *At*4CL1, *Vv*VST	Multiple copy genome integration	*S. cerevisiae* CEN.PK102-5B/Aromatic amino acids overproducing strain	Glucose (40 g/L)	Fed-batch bioreactor	531.4	[60]
**Resveratrol**	*At*PAL, *At*C4H, *At*4CL2, *Vv*VST1	Multiple copy genome integration	*S. cerevisiae* CEN.PK102-5B/Aromatic amino acids overproducing strain; Expression of *At*CPR and CYB5 to enhance cytochrome P450 activity	Glucose (88 g/L)	Fed-batch bioreactor	800	[61]
**Pinostilbene**	*AtPAL, At*C4H*, At*4CL2*, Vv*VST1*, Vv*ROMT					1.96	
**Pterostilbene**	*AtPAL, At*C4H*, At*4CL2*, Vv*VST1, *Vv*ROMT					34.9	
**Pterostilbene**	*At*4CL, *Vv*STS, *Vv*ROMT	Episomal plasmids	*S. cerevisiae* WAT11	Coumaric acid (10 mg/L)	Shake flask	2.2	[62]
**Naringenin chalcone**	*Rt*PAL, *At*4CL1, *Ha*CHS	Episomal plasmids	*S. cerevisiae* AH22/PAD knock-out	Galactose (20 g/L)	Shake flask	7	[63]
**Naringenin**	*At*PAL, *At*CHI, *At*CHS, *At*4CL3, *At*TAL, *At*C4H, *At*CHS3	One copy genome integration	*S. cerevisiae* CEN.PK717.5A/Aromatic amino acids overproducing strain; Expression of *At*CPR to enhance cytochrome P450 activity	Glucose (20 g/L)	Batch bioreactor	108.9	[64]
**Naringenin**	*Ph*PAL, *Gm*4CL2, *Gm*CHS, *Gm*CHI	One copy genome integration	*S. cerevisiae* YPH499/Expression of *Ph*CPR to enhance cytochrome P450 activity	Phenylalanine (1.7 g/L)	Shake flask	8.9	[65]
**Genistein**	*Ph*PAL, *Gm*4CL2, *Gm*CHS, *Gm*CHI, *Gm*IFS					0.1	
**Kaempferol**	*Ph*PAL, *Gm*4CL2, *Gm*CHS, *Gm*CHI, *Gm*F3′H, *St*FLS					1.3	
**Quercetin**	*Ph*PAL, *Gm*C4H, *Gm*4CL2, *Gm*CHS, *Gm*CHI, *Gm*F3′H, *St*FLS					0.26	
**Naringenin**	*Fj*TAL, *Pc*4CL, *Ph*CHS, *Ms*CHI	One copy genome integration	*S. cerevisiae* CEN.PK102-5B/Aromatic amino acids overproducing strain; Expression of *Cr*CPR to enhance cytochrome P450 activity	Synthetic fed-batch medium	Microtiter plates	1.55	[66]
**Liquiritigenin**	*Fj*TAL, *Pc*4CL, *Ph*CHS, *Ms*CHI, *Am*CHR					5.31	
**Kaempferol**	*Fj*TAL, *Pc*4CL, *Ph*CHS, *Ms*CHI, *Am*F3H, *At*FLS					26.57	
**Resokaempferol**	*Fj*TAL, *Pc*4CL, *Ph*CHS, *Am*CHR, *Ms*CHI, *Am*F3H, *At*FLS					0.51	
**Quercetin**	*Fj*TAL, *Pc*4CL, *Ph*CHS, *Ms*CHI, *Am*F3H, *At*FLS, *Ph*FMO					20.38	
**Fisetin**	*Fj*TAL, *Pc*4CL, *Ph*CHS, *Am*CHR, *Ms*CHI, *Am*F3H, *At*FLS, *Ph*FMO					1.65	
**Naringenin**	*Fj*TAL*, At*4CL2, *Ha*CHS, *Ms*CHI	One copy genome integration	*S. cerevisiae* BY4741/Aromatic amino acids overproducing strain; Overexpression of ACC1 and decrease of the flux towards fatty acid biosynthesis	Sucrose (10 g/L)	Shake flask	90	[67]
**Kaempferol**	*Eg*PAL*, Eg*C4H*, Eg*4CL*, Eg*CHS*, Eg*CHI*, At*F3H*, Pd*FLS	One copy genome integration	*S. cerevisiae* W303-1A/Increased flux towards malonyl-CoA synthesis	Glucose (20 g/L)	Fed-batch bioreactor	66.3	[68]
**Naringenin**	*Fj*TAL*, At*4CL1*, Ha*CHS*, Ph*CHI	Episomal plasmids	*S. cerevisiae* BY4741*/*Aromatic amino acids overproducing strain	Glucose (20 g/L)	Shake flask	220	[69]
**Kaempferol**	*Fj*TAL*; At*4CL1*; Ha*CHS*; Cu*F3H*, At*FLS					86	
**Naringenin**	*At*PAL, *At*TAL, *At*C4H, *At*4CL3, *At*CHI, *At*CHS3, *Sf*FPT	One copy genome integration and episomal plasmids	*S. cerevisiae* IMK393/Aromatic amino acids overproducing strain; Expression of *At*CPR to enhance cytochrome P450 activity; replacement of TSC13 by an orthologue gene	Glucose (20 g/L)	Shake flask	100	[70]
**8-Prenylnaringenin**						0.12	
**Liquiritin**	*Gu*PAL*, Gu*C4H*, Gu*4CL1*, Gu*CHS*, Gu*CHR*, Gu*CHI*, Gu*UGT	Episomal plasmids	*S. cerevisiae* WM4-3	Glucose (20 g/L)	Fed-batch bioreactor	0.42	[71]
**Phloterin**	*At*PAL, *Amm*C4H, *At*4CL2, *Ha*CHS, *Sc*TSC13	Episomal plasmids	*S. cerevisiae* BG/Overexpression of *Sc*CPR1 to enhance cytochrome P450 activity	Glucose (20 g/L)	Microtiter plates	42.7	[72]
**Pinocembrin**	*At*PAL, *At*4CL2, *Ha*CHS, *Sc*TSC13					2.6	
**Phlorizin**	*At*PAL, *Amm*C4H, *At*4CL2, *Ha*CHS, *Sc*TSC13, *Pyc*UGT	Episomal plasmids	*S. cerevisiae* BG/Overexpression of *Sc*CPR1 to enhance cytochrome P450 activity	Glucose (20 g/L)	Microtiter plates	65	[72]
**Nothofagin**	*At*PAL, *Amm*C4H, *At*4CL2, *Ha*CHS, *Sc*TSC13, *Os*UGT					59	
**Trilobatin**	*At*PAL, *Amm*C4H, *At*4CL2, *Ha*CHS, *Sc*TSC13, *At*UGT					32.8	
**Naringin dihydrochalcone**	*At*PAL, *Amm*C4H, *At*4CL2, *Ha*CHS, *Sc*TSC13, *At*UGT, *Cm*RHAT, *At*RHM					11.6	
**3-Hydroxyphloretin**	*At*PAL, *Amm*C4H, *At*4CL2, *Ha*CHS, *Sc*TSC13, *Cs*CH3H, *At*ATR					28.8	
**Afzelechin**	*At*PAL*, Anm*C4H*, Ms*CHI*, At*4CL2*, Md*F3H*, Aa*DFR*, Vv*LAR	One copy genome integration	*S. cerevisiae* BG/Expression of *At*CPR1 to enhance cytochrome P450 activity	Glucose (20 g/L)	Microtiter plates	~80 ^4^	[73]
**Catechin**	*At*PAL*, Anm*C4H*, Ms*CHI*, At*4CL2*, Md*F3H*, Md*F3′H, *Aa*DFR*, Vv*LAR					~90 ^4^	
**Gallocatechin**	*At*PAL*, Anm*C4H*, Ms*CHI*, At*4CL2*, Md*F3H*, Md*F3′5′H*, Aa*DFR*, Vv*LAR					~25 ^4^	
**Pelargonidin-3-O-glucosidase**	*At*PAL*, Anm*C4H*, Ms*CHI*, At*4CL2*, Md*F3H*, Aa*DFR*, Ph*ANS*, Dc*A3GT					0.85	
**Cyanidin-3-O-glucosidase**	*At*PAL*, Anm*C4H*, Ms*CHI*, At*4CL2*, Md*F3H*, Md*F3′H*, Aa*DFR*, Ph*ANS*, Fa*A3GT					1.55	
**Delphinidin-3-O-glucosidase**	*At*PAL*, Anm*C4H*, Ms*CHI*, At*4CL2*, Md*F3H*, Md*F3′5´H*, Aa*DFR*, Ph*ANS*, Fa*A3GT					1.86	
**Apigenin**	*At*C4H*, Pc*4CL2*, Peh*CHI*, Peh*CHS*, Pc*FSI	Episomal plasmids	*S. cerevisiae* INVSc1/Overexpression of *Sc*CPR and *Anm*CPR to enhance cytochrome P450 activity	Cinnamic acid (74 mg/L)	Shake flask	0.4	[74]
**Naringenin**	*At*C4H*, Pc*4CL2*, Peh*CHI*, Peh*CHS*, Pc*FSI					0.07	
**Luteolin**	*At*C4H*, Pc*4CL2*, Peh*CHI*, Peh*CHS*, Pc*FSI					1.1	
**Eriodictoyl**	*At*C4H*, Pc*4CL2*, Peh*CHI*, Peh*CHS*, Pc*FSI					2.5	
**Pinocembrin**	*At*C4H*, Pc*4CL2*, Peh*CHI*, Peh*CHS*, Pc*FSI					1.4	
**Naringenin**	*Fj*TAL*, At*4CL1*, At*CHS*, At*CHI	One copy genome integration	*S. cerevisiae* BY4741/Increased flux towards aromatic amino acids and malonyl-CoA biosynthesis	Glucose (20 g/L)	Shake flask	144.1	[75]
**Kaempferol**	*Fj*TAL, *At*4CL1, *At*CHS, *At*CHI, *Nt*F3H*, At*FLS					168.1	
**Quercetin**	*Fj*TAL, *At*4CL1, *At*CHS, *At*CHI, *Nt*F3H*, Ph*F3′H*, At*FLS					154.2	
**Myricetin**	*Fj*TAL, *At*4CL1, *At*CHS, *At*CHI, *Nt*F3H*, Ph*F3′H*, Sl*F3′5′H*, At*FLS					145	
**Pelargodinin**	*Fj*TAL, *At*4CL1, *At*CHS, *At*CHI, *NtF3H, AaDFR, GeANS*					33.3	
**Cyanidin**	*Fj*TAL, *At*4CL1, *At*CHS, *At*CHI, *Nt*F3H*, Ph*F3′H*, Ge*DFR*, Ge*ANS					31.7	
***p*-Coumaroyl-3-hydroxyanthranilic acid**	*Nt*4CL2, *Cc*HCT	Episomal plasmids	*S. cerevisiae* CENPK113-5d/-	Coumaric acid (462 mg/L) and HAA (77 mg/L)	Batch bioreactor	120	[38]
**Caffeoyl-3-hydroxyanthranilic acid**				Caffeic acid (540 mg/L) and HAA (77 mg/L)		22	

^1^ 4CL—4-coumarate-CoA ligase; A3GT—anthocyanidin-3-O-glycosyl transferase; ANS—anthocyanidin synthase; C3H—4-coumarate 3-hydroxylase; C4H—cinnamate-4- hydroxylase; CH3H—chalcone 3-hydroxylase; CHR—chalcone reductase; COMT—caffeic acid O-methyltransferase; CCoAOMT—caffeoyl-CoA O methyltransferase; CHI—chalcone isomerase; CHS—chalcone synthase; CURS—curcumin synthase; CUS—curcuminoid synthase; DCS—diketide-CoA synthase; DFR—dihydroflavonol-4-reductase; F3H—flavanone 3-hydroxylase; F3′H—flavonoid-3′-hydroxylase; F3´5´H—flavonoid-3′,5′-hydroxylase; FLS—flavonol synthase; FMO—cytochrome P450 flavonoid monooxygenases; FPT—flavonoid prenyltransferase; FSI—flavone synthase I; HCT—hydroxycinnamoyl-CoA:quinate hydroxycinnamoyltransferase; HpaB and HpaC—4-hydroxyphenylacetate 3-hydroxylase; IFS—isoflavone synthase; LAR—leucoanthocyanidin reductase; PAL—phenylalanine ammonia lyase; RHAT—flavanone 2-O-rhamnosyltransferase; RHM—rhamnose synthase; ROMT—resveratrol O-methyltransferase; STS—stilbene synthase; TAL—tyrosine ammonia lyase; UGT—isoflavonoid 7-O-glycosyltransferase; VST—resveratrol synthase; *Aa—Anthurium andraeanum; Ah—Arachis hypogaea; Am—Astragalus mongholicus; Amm—Ammi majus; Anm—Antirrhinum majus; At—Arabidopsis thaliana; Cc—Cynara cardunculus; Cm—Citrus maxima; Cr—Catharanthus roseus; Cs—Cosmos sulphureus Cu—Citrus unshiu; Dc—Dianthus caryophyllus; Eg—Erigeron breviscapus; Fa—Fragaria ananassa; Fj—Flavobacterium johnsoniaeu; Ge—Gerbera* specie; *Gm—Glycine max; Gu—Glycyrrhiza uralensis; Ha—Hypericum androsaemum; Hg—Hyperricum androsaemum; Md—Musca domestica; Ms—Mendicago sativa; Nt—Nicotiana tabacum; Os—Oriza sativa; Pa—Pseudomonas aeruginosa; Pc—Petroselinum crispum; Pd—Populus deltoides; Peh—Petunia hybrida; Ph—Populus hybrid; Pyc—Pyrus communis; Rc—Rhodobacter capsulatus; Rt—Rhodosporidium toruloides; Sc—Saccharomyces cerevisiae; Se—Salmonella enterica; Sf—Sophora flavescens; Sl—Solanum lycopersicum; St—Solanum tuberosum; Vv—Vitis vinifera*. ^2^ Chassis modifications in addition to pathway integration when applied. ACC—acetyl-CoA carboxylase; *araE*—arabinose-H^+^ transporter, CPR—cytochrome P450 reductase; PAD—phenylacrylic acid decarboxylase; TAT1—Tyrosine and tryptophan amino acid transporter; CYB5—cytochrome B5; TSC13—double-bond reductase involved in fatty acid synthesis. ^3^ Synthetic fed-batch medium contains a glucose polymer which is converted into glucose monomers by glucose releasing enzymes during the fermentation process; HAA—hydroxyanthranilic acid. ^4^ Values extrapolated from graphics.

#### Genome Integration

Most of the metabolic engineering approaches in *S. cerevisiae* used high copy number 2 micron (2µ) episomal plasmids. In particular, high copy plasmids can be maintained at 10–80 copies per cell [76] providing a convenient platform for the overexpression of heterologous genes. However, there are inherent problems associated with episomal plasmids, namely their instability, variations in gene expression within a population, as well as the high maintenance costs to ensure the selective pressure [77]. The genomic integration of entire biosynthetic pathways appears as an alternative to the use of episomal plasmids since it allows for the stable expression of the heterologous pathways. Nevertheless, integration is unsuitable as an overexpression system because only one copy can be integrated into the yeast genome. Although the multi-copy integration of biochemical pathways is essential, it is still a challenging strategy. The delta integration strategy was developed to achieve the multi-copy integration of heterologous genes by HR at Ty retrotransposon delta sites, a set of repetitive regions in yeast genome. Using this strategy, a heterologous biosynthetic pathway can be inserted in a multi-copy way allowing for the overexpression of recombinant enzymes as it occurs with episomal plasmids [78,79]. For instance, Li et al. [60] used a multiple integrative plasmid to engineer yeast for the de novo biosynthesis of resveratrol. The authors determined the heterologous gene copy number by qPCR. The strain containing multiple copies of the pathway produced 36 times more resveratrol than the strain containing only one copy, although only eight pathway copies were integrated into the yeast genome [60].

**Figure 4:**
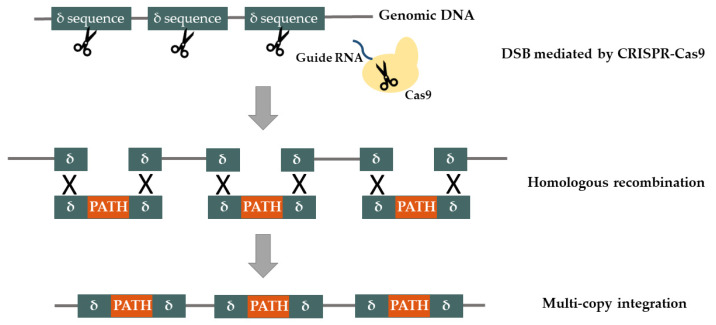
Multi-copy integration of a heterologous pathway at delta sites (δ) mediated by the Clustered Regularly Interspaced Short Palindromic Repeats (CRISPR)-associated caspase 9 endonuclease (Cas9) [63]. DSB—Double strand break.

With the development of new molecular biology techniques, such as the Clustered Regularly Interspaced Short Palindromic Repeats (CRISPR)-associated caspase 9 endonuclease (Cas9) system, it became possible to modify the genome of different microorganisms in a more efficient way. The CRISPR-Cas9 system was first introduced in *S. cerevisiae* by DiCarlo et al. [80]. Nowadays, this system has been routinely used for carrying out high-efficiency gene knock-ins and knock-outs. These modifications are performed without the use of selection markers since the combination of double strand breaks (DSB), caused by CRISPR technology, and the HR capability of yeast has almost total effectiveness. The short DNA donor oligos sharing homology with a target site can serve as the simplest repair template resulting in an efficient HR [80]. Therefore, the combination of CRISPR-Cas9 with HR capability represent a powerful technique to genetically engineer yeast. Shi et al. [81] developed a novel method that combined CRISPR-Cas9 and delta integration (Figure 4). The delta integration CRISPR-Cas9 method is a useful strategy to stably integrate multiple copies of large biochemical pathways without the use of selection markers. Using this strategy, the researchers integrated 18 copies of a 24 kb fragment in the yeast genome in a single step. Although this technique has not been used for polyphenol pathway engineering in yeast, it may be very useful in future experiments.

### 2.2. Pathway Enzyme Engineering

Finding the right enzyme combination is a key step in the genetic engineering of any heterologous pathway. The enzymes for producing polyphenols in an engineered yeast should be carefully selected to ensure a metabolic balance between all the intermediary reactions and consequently obtain higher production yields. Moreover, the use of enzymes that catalyze more than one reaction may decrease the metabolic burden caused to the host, hence resulting in an enhanced production. As previously mentioned, PAL is the first enzyme of the phenylpropanoids pathway. Some PALs also have TAL activity, converting tyrosine directly into *p*-coumaric acid. The use of these enzymes allows the production of *p*-coumaric acid without requiring C4H (a membrane-bound cytochrome P450-dependent enzyme). This represents an advantage since the native cytochrome P450 activity could not be enough to support P450-dependent plant enzymes. Trantas et al. [65] engineered a set of yeasts to produce flavonoids and stilbenoids using phenylalanine as the initial precursor. Depending on the final phenylpropanoid, the constructed pathways contained four to eight enzymatic steps. In addition, the researchers tested if the co-expression of a heterologous cytochrome P450 reductase (CPR) would enhance the activity of the cloned C4H. When the CPR was co-expressed the production of *p*-coumaric acid was enhanced by four times. This indicated that the activity of the endogenous yeast CPR was not sufficient to support the maximum substrates fluxes through C4H. 

The 4CL enzymes family catalyzes the conversion of *p*-coumaric acid, ferulic acid and caffeic acid to the respective CoA esters. Furthermore, 4CL has been shown to occur in multiple isoforms with distinct substrate affinities [82]. In the heterologous production of flavonoids and stilbenoids in *S. cerevisiae*, 4CLs from *Arabidopsis thaliana* are the most commonly used [55,56,83]. *A. thaliana* possesses four isoforms of 4CL (At4CL1, At4CL2, At4CL3 and At4CL4). From the expression patterns and phylogenetic similarity, only At4CL3 is suggested to play a role in flavonoid biosynthesis. At4CL1 and At4CL2 are presumed to be involved in the lignin production [82]. Li et al. [60] evaluated if the use of different *A. thaliana* 4CL isoforms (At4CL1 and At4CL2) would have an impact on the production of resveratrol by the engineered yeasts. The results confirmed that At4CL1 resulted in slightly higher resveratrol titers than At4CL2. These results confirmed that At4CL1 has higher activity towards *p*-coumaric acid as described by Ehlting et al. [82]. Altogether, these results demonstrated that choosing the right enzymes combination is a key step to produce heterologous compounds such as polyphenols in yeast. 

The expression of heterologous genes in *S. cerevisiae* can lead to translation errors, frameshifting events, stalling or premature translational termination due to the existence of rare codons. Consequently, it can result in the expression of a non-functional enzyme. To overcome that, codon optimization must be considered to optimize the heterologous production of polyphenols. For instance, in the work developed by Wang et al. [57], the enzyme TAL from *Rhodobacter sphaeroides* did not show in vivo activity in *S. cerevisiae*. In contrast, the use of a codon-optimized TAL enabled the resveratrol biosynthesis in the engineered yeast.

#### Spatial Location of Heterologous Enzymes

Organelles are important supplies of cofactors. In mitochondria, the pool of acetyl-CoA is higher than in cytoplasm [84]. This may be advantageous for the expression of enzymes related with polyphenol synthesis since malonyl-CoA plays a key role in the biosynthetic pathway. In higher plants, polyphenol biosynthetic enzymes are co-localized and assembled in protein complexes and the metabolic channeling effect decreases the formation of by-products. In analogy, compartmentalization or scaffolding the polyphenol enzymes in yeast may have a positive effect on the enzymatic activity and therefore on the polyphenol production. The compartmentalization of the phenylpropanoid pathway has never been achieved. However, this strategy was already used to produce terpenoids in genetically modified yeasts yielding good results [85]. For this reason, the compartmentalization of the polyphenol pathway appears as a must-try in future works.

Closely locating two active sites or channeling the intermediate compounds between the enzymes may also improve the metabolic efficiency of the heterologous enzymes. This can be accomplished by using fusion proteins or by constructing synthetic scaffolds, respectively. Zhang et al. [86] constructed a translational fusion protein of 4CL from *A. thaliana* and STS from *Vitis vinifera* (4CL:STS). The fusion protein was constructed by replacing the stop codon of the 4CL gene by a three-amino acid linker and the STS sequence. Resveratrol biosynthesis in yeasts transformed with 4CL:STS increased 15-fold as compared with the transformants co-expressing 4CL and STS. Moreover, Wang and Yu [59] constructed synthetic scaffolds to recruit At4CL1 and VvSTS to improve resveratrol production. The scaffolds interacted with the heterologous enzymes through the small peptide ligands that were expressed in the end of each heterologous gene. This strategy allowed the substrate channeling between the two enzymes. The engineered strain produced five-fold more resveratrol over the one where scaffolds were not used. In addition, the production of resveratrol was 2.7-fold improved comparing with the production obtained using fusion proteins [86], thus suggesting more effectiveness when scaffolds are used.

### 2.3. Strain Optimization

The optimization of the heterologous pathways is a key step for the development of efficient yeast cell factories to produce polyphenols. Besides that, the yeast chassis should also be genetically engineered to maximize the production yields. These genetic modifications may include the overexpression or deletion of endogenous genes to increase the polyphenol precursors supply or to prevent the intermediate consumption. These modifications allow the production of polyphenols in higher yields from simple substrates such as glucose, which represents a big step towards an industrial application. In this section, strategies used to improve the yeast chassis for polyphenol production are described.

#### 2.3.1. Amino Acids Overproducing Strains

In order to improve the production of the desired compounds in engineered cells it is important to perform genetic modifications to improve the amounts of precursors available. The modifications could be deletions of genes involved in the deviation of a precursor and consequently, in the production of by-products or the overexpression of genes involved in the precursor biosynthesis. The aromatic amino acids precursors of the phenylpropanoids acids are not produced in high amounts by *S. cerevisiae,* thus representing a limiting step for the polyphenol production [50]. To achieve a high polyphenol yields, the media can be supplemented with precursors such as aromatic amino acids or hydroxycinnamic acids. However, this makes the process expensive for industrial applications. Therefore, the development of strains capable of converting glucose or other simple carbon sources to polyphenols also represents an attractive engineering strategy. The aromatic amino acid biosynthesis is a branched pathway regulated at the transcriptional, translational and allosteric levels. At the allosteric level, the effector molecules are the aromatic amino acids tyrosine and phenylalanine. For example, the reactions catalyzed by CM and 3-deoxy-D-arabino-heptulosonate-7-phosphate synthase (DAHPS), enzymes involved in the shikimate pathway (Figure 2), are feedback-inhibited by tyrosine and phenylalanine [87]. Koopman et al. [64] constructed a mutated *S. cerevisiae* strain capable of overproducing aromatic amino acids by replacing the DAHPS gene, for a feedback-insensitive version. Consequently, the replacement of this gene by an insensitive version was important to maintain the amino acids production. Moreover, the deletion of phenylpyruvate decarboxylase (PDC) that was responsible for the conversion of phenylpyruvate, the precursor of phenylalanine, into phenylacetaldehyde, was important to prevent the pathway deviation. This optimized strain produced larger amounts of naringenin comparatively to the non-optimized strain [64]. In a different study, Li et al. [60] constructed a pathway for the de novo biosynthesis of resveratrol from glucose, and to improve the flux towards aromatic amino acids, the researchers developed a feedback-inhibition-resistant strain overexpressing CM and DAHPS. This resulted in 4.85 mg/L resveratrol, a titer 1.8 times higher than the one obtained without the overexpressed genes [60]. More recently, a strain highly adapted to produce *p*-coumaric acid was developed by performing several modifications in yeast chassis to program the carbon metabolism towards the aromatic amino acid biosynthesis [50]. The heterologous pathway was composed by PAL and C4H from *A. thaliana* and the native cytochrome (CYB5) and the *A. thaliana* P450 reductase were also overexpressed to improve the cytochrome P450 activity. The chassis modifications also included the expression of a feedback insensitive DAHPS and CM. Moreover, three native shikimate pathway genes and a heterologous version of prephenate dehydrogenase (PDH) were overexpressed to increase the total shikimate pathway activity. In order to improve the shikimate precursor E4P availability, a heterologous phosphoketalose-based pathway was inserted. In addition, to optimize the carbon flux from glycolysis towards the aromatic amino acid biosynthesis, promoters of key node genes were replaced. PDC was also deleted to prevent pathway deviation. The final strain produced 12.5 g/L of *p*-coumaric acid, by far the highest titer reported. 

The expression of heterologous metabolic pathways impacts the cellular physiology and frequently triggers a stress response. The identification of the affected pathways and then the genetic modification of target genes can improve the titers of the desired product [88]. Rodriguez et al. [48] found after transcriptome and metabolome computational analyses significant expression changes in a set of genes in an engineered yeast strain able to produce *p*-coumaric acid. Genes involved in the transport of amino acids, polyamines and sugars were found to be downregulated. Then, the researchers knocked out these specific genes to evaluate its effect in *p*-coumaric acid production titers. The deletions improved the synthesis of the polyphenol precursor. The highest improvement (up to 50%) was obtained by the deletion of tyrosine and tryptophan amino acid transporter 1 (TAT1). The authors hypothesized that this deletion resulted in a decrease of the leakage of tyrosine from the cells, thus being more available for the production of *p-*coumaric acid [48].

#### 2.3.2. Extender Substrates Availability

To produce one polyphenol molecule, it is necessary to have at least one molecule of malonyl-CoA since this molecule acts as an extender molecule in the final reactions. However, the levels of malonyl-CoA inside the cell are low, representing a limiting step in the heterologous production of polyphenols. Malonyl-CoA is naturally synthesized by acetyl-CoA carboxylase (ACC). This enzyme catalyzes the carboxylation of acetyl-CoA to malonyl-CoA in microorganisms. The malonyl-CoA is mainly used for the production of fatty acids and phospholipids, leaving only a very limited amount available for the production of secondary metabolites [89].

Shin et al. [58] investigated the production of resveratrol from supplemented tyrosine. They introduced PAL, C4H, 4CL and STS genes into *S. cerevisiae*. In addition, the authors replaced the ACC native promoter by a stronger inducible promoter (pGAL1-galactose inducible promoter). The overexpression of the ACC gene resulted in a 1.3-fold increase in resveratrol concentration [58]. The ACC enzyme can be targeted for degradation via phosphorylation by sucrose non-fermenting protein 1 (Snf1p) in yeast decreasing the endogenous pool of malonyl-CoA inside the cell [90]. Li et al. [60], apart from the aforementioned resveratrol pathway multi-copy integration and DAHPS and CM overexpression, also used a strategy to improve the intracellular malonyl-CoA bioavailability. This strategy included the overexpression of a post-translational deregulated ACC. This version of the ACC gene was previously described by Shi et al. [91] and it contains two-point mutations in serine residues to prevent the phosphorylation and degradation by Snf1p. The engineered strain enhanced the productivity by 31% [60]. Afterwards, the same research team [61], applied a more complex approach. They changed the heterologous pathway to use phenylalanine instead of tyrosine and overexpressed the CPR from *A. thaliana* and the native CYB5 to enhance the P450 function required by C4H. DAHPS and CM were also overexpressed and the PDC was deleted. The shikimate kinase from *E. coli* was also overexpressed to increase the metabolic flux through the shikimate pathway. Regarding the malonyl-CoA bioavailability, in addition to the mutated ACC overexpression, the acetyl-CoA synthase from *Salmonella enterica* was also overexpressed. The final strain produced 800 mg/L of resveratrol in a fed-batch fermentation. This represented by far the highest yield reported regarding the polyphenol heterologous production in *S. cerevisiae*.

Due to the chemodiversity of polyphenols, other extender substrates may be required to obtain the desired final product. With that comes the need to also increase the endogenous production of a given functional group, since its intracellular pool in yeast may not be enough to support the specific polyphenol biosynthesis. For instance, Levisson et al. [70] have optimized a *S. cerevisiae* strain for the de novo production of 8-prenylnaringenin. Initially, an amino acids overproducing strain containing the naringenin biosynthetic pathway integrated into its genome was transformed with an episomal plasmid carrying a prenyltransferase (PT) and a truncated form of 3-methylglutaryl coenzyme A reductase (tHMG1). tHMG1 was expressed to increase the prenyl donor (dimethylallyl diphosphate) supply and consequently, improved the synthesis of 8-prenylnaringenin. However, the production yields were found to be residual. Moreover, the essential gene TSC13 was replaced by the orthologue enoyl-CoA reductase (ECR) gene from *Malus domestica*. TSC13 encodes an enzyme involved in fatty acid synthesis and is able to convert *p*-coumaroyl-CoA into phloretic acid. The replacement of this gene was important to overcome the *p*-coumaroyl-CoA consumption since the ECR gene is not able to convert *p*-coumaroyl-CoA into phloretic acid. Moreover, the TAL gene was integrated into the genome of this strain to increase the production by using the two aromatic amino acids as precursors. Before integrating the TAL gene, this strain was only using the phenylalanine route to produce the desired compound. These modifications resulted in a five-fold improvement of naringenin and 10-fold improvement of 8-prenylnaringenin productions. 

#### 2.3.3. Transporter Expression

The transport of small molecules into and out of the cell can be sometimes the biggest limiting factor in metabolic engineering. Transporters can maximize the productivity by improving substrate uptake or product secretion. The removal of a product with reported antimicrobial activity from the intracellular medium will increase the product biosynthesis and decrease the product toxicity for the cell and the extraction costs. Polyphenols are characterized to often accumulate in plant vacuoles [92] and afterwards be transported to the site of action. However, the mechanism of polyphenol transportation is not yet fully understood. Wang et al. [57] expressed the arabinose-H+ transport protein (AraE) in a resveratrol engineered yeast. The transporter greatly improved the resveratrol synthesis. This suggests that AraE may increase the resveratrol permeability through lipid membranes when the concentration inside the cell reaches the threshold. More studies on polyphenol transport are required, not only to increase their production but also to enable their extraction as soon as an industrial production process is implemented. 

## 3. Key Messages

In recent years, the demand for polyphenols by the pharmaceutical industry has increased. Consequently, the scientific community has been developing several efforts to establish a stable platform for the production of these therapeutic compounds. The use of microbial cell factories is an attractive approach due to the low costs, the short production times and the high purity when compared to the plant extraction. The advances in the synthetic biology and metabolic engineering fields allowed the creation of new and powerful genetic tools. The use of these tools in conjugation with the development of computational omics is enabling the genetic engineers to construct host chassis increasingly adapted to the production of polyphenols. So far, the different synthetic biology approaches used by several researchers have showed significant improvements in the production of these compounds using engineered *S. cerevisiae*. Nevertheless, the implementation of an industrial process to produce polyphenols using microbial cell factories still has a long way to go before becoming a reality. However, the recently high production of the precursor *p*-coumaric acid obtained in *S. cerevisiae* [50] opened the door to producing high amounts of polyphenols in the near future. In addition, some polyphenols, such as curcuminoids and coumarins, have never been produced in *S. cerevisiae* although the pathways are generally well characterized and have been successfully produced in *E. coli* [39,93]. We believe that these pathways can be effectively expressed in yeast, possibly with higher titers, and therefore should be explored. Overall, this review provided an overview of diverse recent advances in the heterologous production of polyphenols in *S. cerevisiae*. Moreover, an outline of different synthetic biology methodologies that can be useful to further optimize *S. cerevisiae* towards the production of high amounts of several polyphenols was herein provided. In summary, we believe that after several optimizations of the host chassis, large amounts of polyphenols could be industrially produced in a fast, efficient and environmentally friendly process.

## Figures and Tables

**Table 1:** Biological activities of some polyphenols.

Biological Activity	Mechanism of Action	References
Anticancer	Curcumin and quercetin reduce the number and size of adenomas in patients with familial adenomatous polyposis. Curcumin treatment in patients with colorectal cancer improves the expression of p53 tumor suppressor and modulates the expression of the apoptosis-related molecules Bax and Bcl-2 inducing cells apoptosis. Curcumin and quercetin modulate the breast cancer type 1 susceptibility protein (BRCA1) levels and inhibit the migration and survival of triple negative breast cancer cells. Resveratrol inhibits proliferation-related proteins and cell proliferation in nasopharyngeal carcinoma cells. Quercetin increases FasL mRNA expression and p51, p21 and growth arrest and DNA damage-inducible 45 proteins signaling activities inducing apoptosis and cell cycle arrestment in triple negative breast cancer cells. Proanthocyanidins modulate the expression of miRNA inhibiting the proliferation of pancreatic cancer cells. Naringenin eliminates the migration and evasion of glioblastoma cells through the inhibition of matrix metalloproteinases, estrogen receptor (ER) and p38 activities and the modulation of epithelial–mesenchymal transition markers.	[18,19,20,21,22]
Anti- inflammatory	Curcuminoid–piperine administration in patients with metabolic syndrome decreases C-reactive protein concentrations in plasma. Naringenin inhibits both inflammatory pain and neurogenic inflammation. The mechanism involves the inhibition of carrageenan-induced oxidative stress, hyperalgesic cytokines production and nuclear factor kappa- B activation. Curcumin increases the production of anti-inflammatory cytokines in microglial cells.	[23,24,25]
Antiviral	Genistein blocks a late-phase event in the life cycle replication of Herpes B virus reducing virus replication and spread. Resveratrol inhibits the viral multiplication of pseudorabies virus in host cells.	[26,27]
Antimicrobial	Polyphenols exhibit antimicrobial effects against both Gram-negative and Gram-positive bacteria. The destabilization of the outer membrane of Gram-negative microorganisms, as well as interactions with the cell membrane might be one of the specific mechanisms behind the antibacterial action. Several polyphenols suppress microbial virulence factors. The reduction of host ligands adhesion, inhibition of biofilm formation and the neutralization of bacterial toxins has been demonstrated. In addition, a synergism between polyphenol and antibiotics has been observed.	[28,29]
Anti-ageing	Resveratrol administration in patients with mild–moderate Alzheimer’s disease decreases matrix metalloproteinase 9 levels and induces immune responses that increase the brain resistance to amyloid precursor protein/β-amyloid deposition. Curcumin administration in non-demented adults results in memory and attention improvements due to the lower accumulation of amyloid and tau in the brain. Orally administered flavonoid rutin significantly attenuates memory deficits in Alzheimer’s disease in transgenic mice by interaction with amyloid β peptides.	[30,31,32]
Estrogenic	8-prenylnaringenin administration in post-menopausal women exhibits positive effects in the reduction of menopausal discomforts. 8-prenylnaringenin promotes bone formation and inhibits the loss of bone density by interacting with ERα in postmenopausal osteoporosis. These effects are more potent than the effects of other phytoestrogens (genistein and daidzein).	[33,34]
